# DNA Methylation as a Programmable Information Layer: From Molecular Marks to Disease State Engineering

**DOI:** 10.3390/ijms27167077

**Published:** 2026-08-07

**Authors:** Lin Du, Yanan Dong, Jin Yang, Zimeng Zhang, Ziyu Liu

**Affiliations:** Beijing Advanced Innovation Centre for Biomedical Engineering, School of Engineering Medicine, Beihang University, Beijing 100191, China; 23101044@buaa.edu.cn (L.D.); yanandong1231@buaa.edu.cn (Y.D.); yyangjin@buaa.edu.cn (J.Y.); zy2310230@buaa.edu.cn (Z.Z.)

**Keywords:** DNA methylation, epigenome editing, epigenetic clocks, cfDNA biomarkers, CRISPR-dCas9, programmable epigenetics

## Abstract

DNA methylation has long been regarded as a stable, maintenance-based epigenetic marker. However, this classical binary model struggles to fully explain the dynamic and situational dependence of methylation regulation at the multi-biological level. This review defines DNA methylation as a programmable information layer that systematically integrates the latest advances in three interrelated dimensions of molecular coding, disease status indication, and epigenomic engineering. At the molecular level, this paper describes how the chemical diversity of cytosine modification, the writing–erasing enzyme network, and the three-dimensional structure of chromatin jointly construct a methylated polymorphic coding system and evaluates the performance of emerging sequencing technologies in DNA integrity, reading length, modification resolution, and analytical complexity through a multidimensional scoring framework. At the cellular and clinical levels, this paper comprehensively demonstrates methylation as a quantifiable indicator of cell identity, biological aging and disease status, covering circulating free DNA biomarkers and spatial heterogeneity analysis. Critically, this paper evaluates how the clustered regularly interspaced short palindromic repeats (CRISPR)-based epigenome editing platform achieves causal inference and promotes the transformation of methylation from related biomarkers to functional therapeutic targets. At the same time, persistent challenges such as off-target specificity, in vivo delivery, and spatiotemporal regulation encountered in epigenetic gene editing are discussed. This review reveals the paradigm shift of DNA methylation from passive observation markers to actively engineered regulatory parameters, which has direct therapeutic application prospects.

## 1. Introduction

DNA methylation is one of the most extensively studied epigenetic modifications in mammalian genomes. Together with histone and other DNA-binding protein remodeling, it forms epigenetic modifications that can alter chromatin structure, silence specific genes, and participate in transcriptional repression, thereby establishing stable gene expression profiles [1]. The classical model of mammalian CpG sites primarily describes DNA methylation as a maintenance-based epigenetic mechanism, where methylation patterns established during development are faithfully transmitted during DNA replication by maintenance DNA methyltransferases such as DNA methyltransferase 1 (DNMT1) [2].

Under this idealized stable inheritance, the classical model implies that all cells within the same tissue exhibit identical methylation patterns. DNA methylation plasticity and epigenetic reprogramming have been recognized for decades, while genome-wide methylation analyses and functional epigenomic studies have further clarified their extent and context dependence [3]. Therefore, DNA methylation is not a simple binary regulatory system, but a dynamic regulatory system influenced by environmental exposure, aging, enzymatic activity, metabolic state, and genetic alterations [4,5,6,7]. A landmark demonstration of epigenetic plasticity came from the induction of pluripotent stem cells using the defined transcription factors OCT3/4, SOX2, KLF4, and c-MYC. This work showed that differentiated somatic cells can undergo extensive epigenetic resetting and return to a pluripotent state, establishing cellular reprogramming as a central framework for studying the reversibility of DNA methylation and cell identity [8,9].

At the molecular level, DNA methylation patterns are regulated by chemical modifications of cytosine residues [10]. At the cellular level, DNA methylation during embryonic development contributes to the establishment and maintenance of cell identity by receiving environmental signals and regulating developmental genes [11]. At the organismal level, methylation patterns undergo continuous changes alongside aging, immune activation, metabolic adaptation, and disease progression, providing molecular readouts of the organism’s biological state [12]. Abnormal methylation signatures have been associated with the development of cancers, cardiovascular disorders, immune dysfunctions, and neurological disorders [13,14,15]. Efforts have demonstrated that the methylation patterns of free DNA in blood can serve as highly sensitive diagnostic biomarkers for disease detection and monitoring [16,17]. Consequently, therapeutic interventions targeting epigenetic dysregulation play a pivotal role in human pathology.

**Figure 1:**
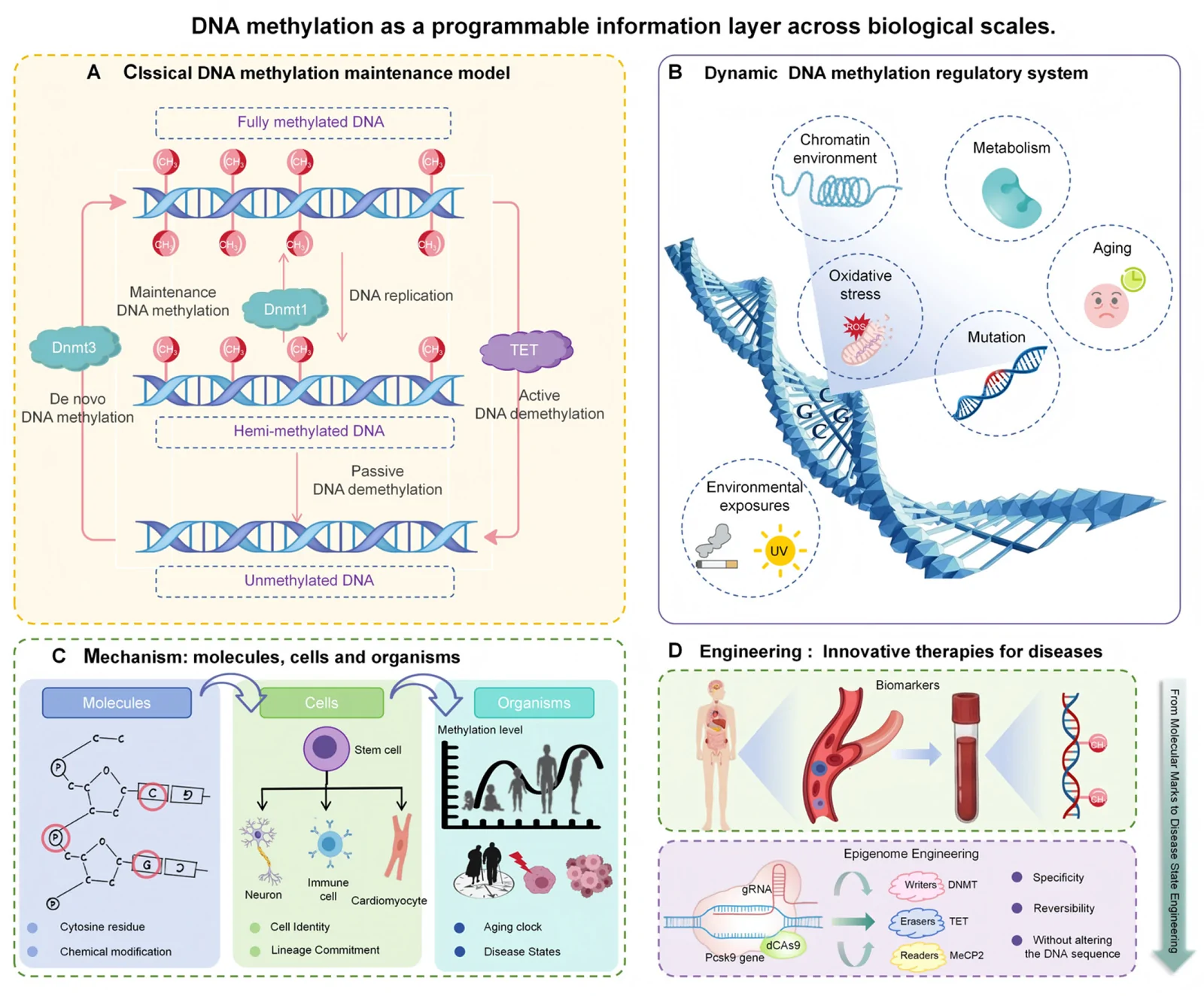
DNA methylation as a programmable information layer across biological scales. Panel (**A**) illustrates the classical DNA methylation maintenance model: de novo methyltransferase DNMT3 fully methylates unmethylated DNA; maintenance methyltransferase DNMT1 transfers the methylation during DNA replication. Panel (**B**) illustrates the dynamic regulatory system: DNA methylation landscapes are shaped by multiple intrinsic and extrinsic factors, including chromatin context, metabolic state, oxidative stress, environmental exposures, aging, and genetic mutations. Panel (**C**) demonstrates multi-scale biological interactions at the molecular, cellular, and organismal levels. Cytosine methylation, as a chemical modification at the molecular level, is associated with cellular identity and the overall state of an organism. Panel (**D**) illustrates the engineering of epigenetic modifications: the characteristics of DNA methylation have shifted from serving as disease biomarkers to being programmable for targeted regulation. Therefore, site-specific regulation of epigenetic states can be achieved without altering the DNA sequence. The figure was designed and assembled by the authors. Some graphical elements and icons were obtained from MedPeer (https://medpeer.cn/) and Iconfont (https://www.iconfont.cn/).

Importantly, technological advances are transforming DNA methylation from a descriptive biomarker into a programmable regulatory layer. For example, CRISPR-based epigenome editing approaches allow site-specific installation of epigenetic memory, thereby achieving direct manipulation of transcriptional states [18]. Together with emerging analytical tools for epigenomic mapping and interpretation [19], these developments support a new conceptual framework in which DNA methylation functions as a multi-scale information layer linking molecular marks to cellular states and disease phenotypes. Therefore, understanding how this epigenetic information is encoded, interpreted, and engineered is crucial for innovative therapies in various diseases, including cancer. The conceptual framework linking DNA methylation across molecular, cellular, organismal, and engineering scales is illustrated in Figure 1.

## 2. Molecular Encoding and Decoding of DNA Methylation

### 2.1. Chemical Diversity of Cytosine Modification

In the mammalian genome, cytosine modification should not be reduced to a binary distinction between methylated and unmethylated cytosines. Ten-eleven translocation (TET) enzymes oxidize 5-methylcytosine (5-mC) into 5-hydroxymethylcytosine (5-hmC), 5-formylcytosine (5-fC), and 5-carboxylcytosine (5-caC), generating a group of chemically related cytosine states with different functional properties [20,21]. Together, these modifications expand the chemical states that cytosine can occupy and change how DNA methylation is described. 5-mC is usually treated as the more stable and heritable mark. 5-hmC, 5-fC, and 5-caC typically reflect local methylation statuses, which are being read, remodeled, or prepared for removal [22]. For example, 5-hmC is significantly enriched in the neuronal genome. It binds to MeCP2 and correlates with active genomic regions, which indicates its role as a distinct regulatory mark [23]. 5-fC and 5-caC at a single hemi-modified CpG site are sufficient to reverse transcriptional repression within specific promoter contexts despite their low abundances, suggesting that even rare modifications may participate in local gene regulation [24].

In mouse embryonic neural stem cells, TET deficiency disrupts glial gene expression and cortical development, indicating that cytosine oxidation contributes directly to lineage competence [25]. 5-hmC in early human embryos is enriched at OTX2-bound enhancers, where it may participate in zygotic genome activation and early lineage specification [26]. Cytosine-state diversity also appears at the level of individual CpG strands. Varying modifications may occur on opposite DNA strands, generating asymmetric or hemi-modified CpG configurations and thereby increasing the potential information carried by a single CpG site [27]. These findings move the discussion beyond the presence or absence of methylation at a CpG site.

The chemical diversity of cytosine modification complicates the classical maintenance model of DNA methylation. In the classical model, the copy mechanism mediated by DNMT1 after DNA replication explains how the 5-methylcytosine (5-mC) pattern can be retained in stable somatic cells. However, this mechanism cannot fully explain the flexibility conferred by TET oxidation and developmental reprogramming processes [3,28]. During embryonic development, primordial germ cell formation, and somatic differentiation, methylation landscapes are frequently erased and rebuilt [29]. Different cytosine states expand the chemical information available at individual CpG sites. Their regulatory meaning depends on genomic position, developmental timing, and strand-specific configuration.

### 2.2. Enzymatic Control of Methylation Dynamics

It is currently believed that the clearance, reconstruction, and maintenance of DNA methylation all rely on the participation of specific enzymes. DNA methyltransferase 3A (DNMT3A) and DNA methyltransferase 3B (DNMT3B) are responsible for establishing de novo methylation in previously unmodified genomic regions, while DNMT1 and ubiquitin-like with PHD and RING finger domains 1 (UHRF1) maintain the methylation pattern after DNA replication [6,30,31]. UHRF1 can recruit DNMT1 to replication-related condensates through liquid–liquid phase separation. It thus confines methylation inheritance to specific local nuclear compartments, transcending the scope of a simple enzyme–substrate reaction [32]. On the demethylation side, TET enzymes oxidize 5-mC, whose oxidized bases can be removed through thymine DNA glycosylase (TDG)-mediated base-excision repair (BER) pathways to regenerate unmodified cytosine [33]. Methylation dynamics mediated by Tet may also influence higher-order chromatin organization. This effect becomes evident upon Tet inactivation, which weakens compartmentalization, topologically associating domain (TAD) structure, and long-range chromatin loops through altered methylation in CpG island (CGI) domains and CCCTC-binding factor (CTCF)-associated regions [34]. During development, aging, and disease progression, the activities of methylation writers, maintainers, and erasers are continually adjusted [35,36,37], producing regional erasure, reconstruction, and boundary changes that contribute to stage-specific and cell-state-specific patterns [38,39].

Metabolite availability is another essential factor in the enzymatic cycle of DNA methylation, which can regulate the speed and success of enzymatic reaction. DNMT enzymes use S-adenosylmethionine as the methyl donor, while TET enzymes require Fe(II) and α-ketoglutarate [40,41]. Mitochondrial metabolism can directly participate in chromatin regulation and DNA methylation, since α-ketoglutarate and related metabolites are supplied through the tricarboxylic acid cycle (TCA cycle) [42]. In pluripotent cells, a high ratio of α-ketoglutarate to succinate supports active demethylation and helps preserve chromatin states related to pluripotency. This link is disrupted in cells with *IDH1/2* mutations. These mutations promote the production of 2-hydroxyglutarate, which inhibits α-ketoglutarate dependent dioxygenases, including TET enzymes, and contributes to hypermethylation phenotypes [43,44,45]. One-carbon and methionine metabolism regulate SAM supply and methylation adaptation to specific regions [46,47].

**Figure 2:**
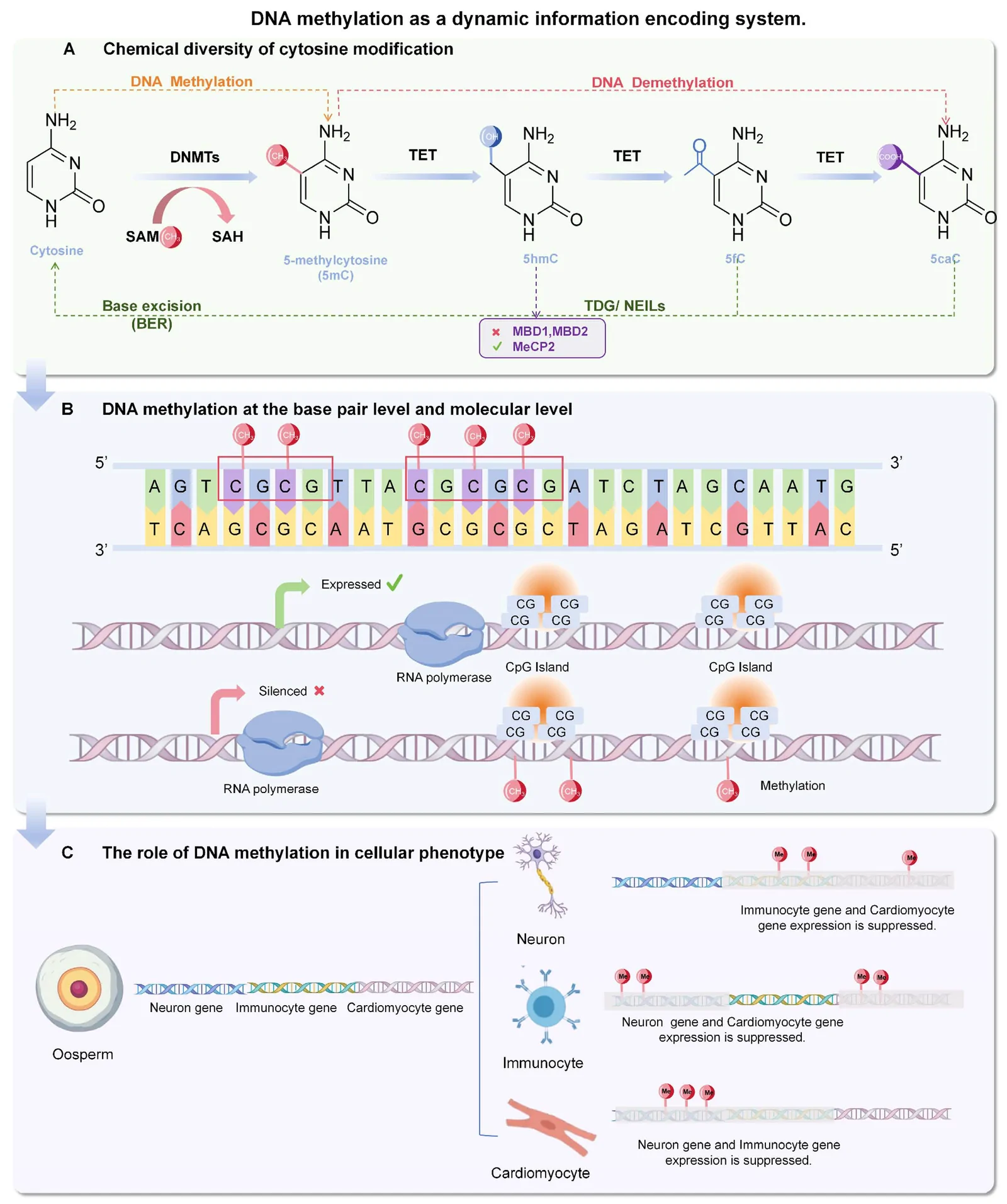
DNA methylation as a dynamic information encoding system. Panel (**A**) illustrates that cytosine undergoes dynamic methylation and demethylation through DNMT-, TET-, and TDG/BER-mediated pathways, generating multiple functional cytosine states, including 5mC, 5hmC, 5fC, and 5caC. Panel (**B**) illustrates that DNA methylation at CpG sites regulates transcriptional activity, with unmethylated CpG islands generally associated with active transcription and methylated regions linked to gene silencing. Panel (**C**) demonstrates that cell-type-specific methylation patterns contribute to lineage specification and maintenance by repressing inappropriate gene expression profiles in different differentiated cell types. The figure was designed and assembled by the authors. Some graphical elements and icons were obtained from MedPeer (https://medpeer.cn/) and Iconfont (https://www.iconfont.cn/).

DNA methylation can function as durable epigenetic memory in tissue homeostasis, but this durability depends on active enzymatic maintenance. Its behavior becomes more dependent on context during embryonic development, cell fate transitions, and pathological reprogramming [48,49,50]. Lu et al. illustrated this balance between stability and reversibility by showing that OSK restored youthful methylation and functional features in retinal ganglion cells through TET1/2-dependent demethylation, without broadly erasing somatic identity [51]. The balance between replication-coupled maintenance, active demethylation, and metabolic input determines when methylation states are preserved, remodeled, or erased. The enzymatic conversion of cytosine states and the roles of DNA methylation in transcriptional regulation and cell identity are illustrated in Figure 2.

### 2.3. Chromatin Context and Regulatory Constraints

DNA methylation is regulated by enzymes, which function within the constraints set by local chromatin structure [52]. Nucleosome positioning can block access to CpG sites. This helps explain why methyltransferases and demethylation enzymes do not remodel DNA uniformly across the genome [53,54]. Remodelers such as LSH/HELLS alter nucleosome positioning and local accessibility, making additional genomic sites available to methylation machinery [55]. CDCA7-HELLS recognizes hemimethylated CpGs within nucleosomes, showing that maintenance-related methylation machinery can act on nucleosomal DNA itself. DNMT3A-DNMT3L similarly place de novo methyltransferase activity in specific chromatin contexts [56,57]. These findings restrict enzyme access in some regions and create local platforms for methylation enzyme recruitment in others.

Histone modifications also help decide where methyltransferases can act. At active promoters, H3K4me3 keeps DNMT3A/B from being activated through the ADD domain, so transcriptionally active regions are less likely to acquire inappropriate methylation [58]. By contrast, methylation at H3K36me3 and H3K36me2/3 helps position DNMT3B and DNMT3A at selected regulatory regions in a cell-state-dependent manner [59,60]. DNMT3A1 can recognize H2AK119ub1-modified nucleosomes, linking DNA methylation with Polycomb-associated regulation [61]. In the DNMT3A2-DNMT3L structure, the Pro-Trp-Trp-Pro (PWWP) domain forms a trilateral interface with the ATRX-DNMT3-DNMT3L (ADD) and methyltransferase domains. This interface masks the H3K36me2-binding pocket, the H3K4me0-binding site, and the target recognition domain (TRD) CpG-recognition loop; H3K36me2 binding and H3K4me0 recognition then release different tiers of autoinhibition and permit catalytic engagement with appropriate chromatin [62]. Thus, histone marks contribute to determining where methyltransferase activity is blocked, recruited, or activated.

Chromatin constraints can also be remodeled during cell fate transitions, development, and disease progression [63]. Pioneer transcription factors are especially important because they can access nucleosomal DNA and create local chromatin openings. FOXA1 binds nucleosomal DNA to initiate chromatin opening, providing access for subsequent regulatory factors [64], which enables small-molecule ligands to reprogram FOXA1 binding and redistribute chromatin accessibility and methylation patterns [65]. Similarly, GATA3-mediated remodeling requires coordinated recruitment of activator protein 1 (AP-1) and switch/sucrose non-fermentable (SWI/SNF), suggesting that pioneer-factor activity depends on cooperative chromatin remodeling [66]. Chromatin context acts as both a constraint and a regulatory guide for DNA methylation. Nucleosome positioning can restrict methyltransferase activity, while histone marks affect whether methyltransferases are recruited or kept inactive. Regions that are initially closed may also become available after pioneer-factor-mediated remodeling. These mechanisms place DNA methylation patterns within the chromatin architecture where methylation enzymes operate.

### 2.4. Advanced Technologies for Methylation Decoding

Advances in sequencing and molecular profiling have substantially changed the detection of DNA methylation. Traditional bisulfite-based methods remain important, yet their use is constrained by DNA degradation, limited discrimination among cytosine modifications, and the loss of broader genomic context. Newer methods are designed to preserve native DNA, distinguish cytosine modifications with higher specificity, and read methylation patterns across broader genomic regions. To compare these methods, we used four evaluation dimensions: DNA integrity and fidelity (DIF), read length and context (RLC), modification resolution (MR), and protocol and analysis simplicity (PAS). These dimensions assess DNA preservation, contextual information provided by sequencing reads, the ability to resolve cytosine modifications, and overall workflow complexity, respectively. The detailed scoring criteria are presented in Table 1.

Conversion-based technologies remain important for methylation decoding because they are compatible with established short-read sequencing workflows. However, conventional bisulfite sequencing cannot distinguish 5-mC from 5-hmC because both modifications resist deamination. Its harsh reaction conditions can also cause substantial DNA degradation and sequence bias [67]. These limitations have motivated the development of alternative conversion-based approaches that improve DNA preservation and modification specificity. Ultrafast bisulfite sequencing (UBS-seq) reduces treatment time and supports methylation profiling from low-input materials, including rare cells and cfDNA [68]. However, UBS-seq still relies on destructive bisulfite chemistry and cannot fully distinguish diverse cytosine modification states [69]. Enzymatic Methyl-seq (EM-seq) improves DNA preservation by replacing bisulfite treatment with TET2 oxidation, T4 bacteriophage beta-glucosyltransferase (T4-BGT)-mediated protection, and apolipoprotein B mRNA editing enzyme, catalytic polypeptide-like (APOBEC)-mediated deamination. It can maintain relatively uniform guanine-cytosine (GC) coverage and high library complexity with as little as 100 pg of starting DNA [70]. TET-assisted pyridine borane sequencing (TAPS) further avoids harsh deamination conditions through TET-assisted chemical conversion, which improves sequencing quality and DNA recovery [71]. Due to their underlying chemical principles, however, standard EM-seq and TAPS still combine 5-mC and 5-hmC signals. beta-glucosyltransferase-assisted TAPS (TAPSβ) introduces β-glucosyltransferase-mediated protection of 5-hmC and enables single-base deconvolution of these two modifications [72]. Higher modification resolution therefore improves the interpretability of methylation profiles, although it usually requires additional enzymatic reactions and more complex experimental workflows.

Native long-read sequencing offers a complementary strategy by preserving methylation information on relatively intact DNA molecules. Single-molecule real-time (SMRT) sequencing records polymerase kinetic signals during nucleotide incorporation, mainly interpulse duration and pulse width, allowing base modifications to be inferred without bisulfite conversion [73]. Circular consensus sequencing (CCS) improves long-read accuracy while retaining modification-sensitive kinetic information [74,75]. Ni et al. developed ccsmeth for single-molecule 5-mCpG detection from long CCS reads of at least 10 kb. The method uses deep learning models, including Bidirectional Gated Recurrent Units, and achieved an accuracy of 0.90 with an area under the receiver operating characteristic curve (AUC) of 0.97. At 10× sequencing depth, ccsmeth showed a genome-wide correlation above 0.90 with bisulfite sequencing and supported haplotype phasing and allele-specific methylation analysis in complex genomic regions [76]. Here, accuracy refers to the proportion of correctly classified single-molecule 5-mCpG calls in the validation datasets used in the original study, which included samples with known methylation status. AUC reflects the ability of the model to distinguish methylated from unmethylated CpG sites across classification thresholds. The reported genome-wide correlation, by contrast, evaluates site-level methylation-frequency concordance with reference methylation profiles at a given sequencing depth, rather than read-level classification accuracy. Oxford Nanopore Technologies (ONT) sequencing similarly analyzes unamplified DNA molecules and detects methylation through electrical current changes as DNA passes through nanopores [77]. These native long-read methods can link methylation patterns with sequence variation, structural variants, haplotypes, and allele-specific information on the same DNA molecule. This feature is especially useful in repetitive sequences, imprinted loci, and structurally complex genomic intervals that are difficult to resolve with short-read approaches [78,79]. Their performance remains affected by sequencing depth, weak modification-associated signals, electrical noise, and the accuracy of basecalling and methylation-calling models [80,81,82].

Targeted and integrative long-read strategies further extend methylation analysis by connecting epigenetic information with selected loci or additional regulatory features. Cas9-targeted nanopore sequencing (nCATS) enriches predefined genomic regions and allows single-nucleotide variants, structural variants, and CpG methylation to be profiled on the same molecule [83,84]. Its targeted design increases local sequencing coverage, although it limits unbiased genome-wide discovery. Whole-genome long-read TAPS (wglrTAPS) combines low-damage TAPS chemistry with PacBio long-read sequencing. At a sequencing depth of 8.2×, wglrTAPS achieved an N50 read length of 3.5 kb, a conversion rate of 96.23%, and a false-positive rate of 0.36%. It enabled methylation profiling across repetitive elements, structural variants, and allele-specific regions that are difficult to examine using short-read TAPS [85]. Although maintaining uniform conversion across long DNA molecules remains technically challenging and may still affect DNA integrity [86], long-read sequencing continues to expand the scope of methylation analysis. In addition to improving genomic coverage, nanopore-based single-molecule multi-omic approaches can simultaneously profile chromatin accessibility and DNA methylation on individual DNA molecules, linking methylation status with local regulatory context [87,88].

Dedicated bioinformatic workflows are required to translate long-read methylation signals into interpretable regulatory evidence. For Oxford Nanopore datasets, Dorado can generate sequence and modified-base calls, Remora supports the development and evaluation of signal-level modified-base models, and Modkit enables post-processing of modification-tagged binary alignment/map (BAM) files into site-level methylation summaries. Independent neural-network frameworks, including DeepSignal and DeepMod2, further support methylation calling from nanopore electrical signals, whereas Methylartist facilitates locus-level visualization and inspection of modified-base profiles [89,90,91,92,93,94]. For PacBio CCS datasets, kinetic-signal processing and haplotype-aware methylation analysis should be performed using platform-specific workflows, such as the ccsmeth and ccsmethphase framework described above. Quality control should assess read length, alignment quality, CpG coverage, modification-calling confidence, model version, and sequencing chemistry because methylation estimates can vary across callers, platforms, and genomic contexts [95,96]. Retaining read-level information is essential for quantifying single-molecule methylation heterogeneity, linking methylation to nearby sequence variants, and resolving allele-specific methylation. NanoMethPhase and MethPhaser illustrate workflows that combine methylation information with long-read variant calling and haplotype phasing [97,98]. Reproducible comparison across studies also requires harmonized reference assemblies, software versions, filtering rules, coverage thresholds, confidence cutoffs, and batch-effect assessment. Reference datasets generated across laboratories and methylation protocols can support the benchmarking and standardization of these analytical decisions [99]. Finally, integration of haplotype-resolved methylation with chromatin accessibility, full-length transcript profiles, and genetic variation can distinguish mechanistically informative regulatory events from correlative methylation signatures and prioritize loci for functional validation [100]. The detection principles, technical strengths, evaluation scores, and typical applications of representative DNA methylation profiling technologies are compared in Table 2.

**Table 2:** Technical comparison of sequencing and conversion-based DNA methylation profiling methods.

Technology	Detection Principle	Key Strengths	Unique Capability	Evaluation Metrics	Total Score	Typical Applications	References
UBS-seq	Ultrafast bisulfite chemical conversion	Reduced DNA damage; high conversion efficiency	Works with ultra-low input samples	[DIF:1.5] [RLC:1.0] [MR:1.0] [PAS:3.0]	6.5	cfDNA; rare cell populations	[67,68,69]
EM-seq	TET oxidation + APOBEC enzymatic deamination	Low DNA damage; uniform coverage	Effective with picogram DNA input	[DIF:2.5] [RLC:1.0] [MR:1.5] [PAS:2.5]	7.5	Low-input epigenomics	[70]
TAPS	TET oxidation + pyridine borane reduction	Bisulfite-free; high sequencing quality	Reduced systematic bias	[DIF:2.5] [RLC:1.0] [MR:1.5] [PAS:2.5]	7.5	Whole-genome methylation analysis	[71]
TAPSβ	βGT protection of 5-hmC before TAPS	Distinguishes 5-mC and 5-hmC	Single-base modification deconvolution	[DIF:2.5] [RLC:1.0] [MR:3.0] [PAS:1.5]	8	Cytosine modification diversity studies	[72]
SMRT-CCS/ccsmeth	Polymerase kinetic signals (interpulse duration (IPD) and pulse width (PW)) on long CCS reads	High-accuracy long reads; direct detection; haplotype phasing	Single-molecule 5-mCpG calling with deep learning	[DIF:3.0] [RLC:3.0] [MR:2.0] [PAS:1.5]	9.5	Allele-specific methylation; complex genomic regions	[73,74,75,76,82]
Nanopore sequencing	Electrical current disruption during DNA translocation	Ultra-long reads; PCR-free; direct detection	Simultaneous SNV, SV, and methylation profiling	[DIF:3.0] [RLC:3.0] [MR:2.5] [PAS:1.5]	10.0	Repetitive regions; clinical methylome profiling	[77]
nCATS	Cas9-targeted nanopore enrichment	Targeted deep coverage; native methylation preserved	Multi-variant detection within same molecule	[DIF:3.0] [RLC:2.5] [MR:2.5] [PAS:1.5]	9.5	Disease-associated loci; imprinting studies	[83,84]
wglrTAPS	TAPS chemistry + long-read sequencing	Long-range methylation phasing	Resolves repetitive and structural variant regions	[DIF:2.5] [RLC:3.0] [MR:2.5] [PAS:1.0]	9	Allele-specific methylation; centromeres	[79,85,86]
Long-read multi-omic integration	Single-molecule joint profiling	Continuous molecular decoding	Co-localization of accessibility and methylation	[DIF:3.0] [RLC:3.0] [MR:3.0] [PAS:0.5]	9.5	Chromatin structure studies	[87,88]

## 3. DNA Methylation as a Cellular and Disease State Indicator

### 3.1. Methylation Patterns and Cell Identity

DNA methylation is closely tied to cell identity. Its patterns retain lineage information but remain adjustable during cell fate transitions [101]. On promoters, enhancers, and other regulatory elements, lineage-specific methylation differences affect chromatin accessibility and transcriptional activity [102,103], thereby cooperatively maintaining cell type-specific gene expression profiles and inhibiting the activation of inappropriate lineage programs [104,105].

Large-scale reference epigenome maps reveal that different cell types have distinct regulatory methylation landscapes, which makes methylation patterns useful as cell-state fingerprints [106]. At the *IL1RN* promoter, targeted methylation editing shifts human myeloid cell identity and inflammatory responsiveness [107]. The SpliCOOL-seq reads DNA methylation together with chromatin accessibility, enabling cell types and tumor subclones to be separated by their epigenetic profiles [108]. Some methylation memory can also survive reprogramming. induced pluripotent stem cells (iPSCs) may retain aberrant methylation hotspots, and transdifferentiated cells can preserve development-specific traces from their original identity [109,110]. These results suggest that DNA methylation acts both as a cell-state fingerprint and as a regulatory barrier that influences how completely cell identity can be reset.

Cell types differ in reproducible CpG methylation signatures, which makes it possible to estimate cellular composition from bulk methylation profiles [111]. In epigenome-wide association studies, this correction is essential, since shifts in cell-type composition may otherwise be mistaken for methylation changes associated with the phenotype of interest [112]. Reference studies of purified blood cells further confirmed that many methylation differences are cell-type-specific and suitable for distinguishing major immune cell populations [113]. In circulating DNA, tissue-specific methylation patterns can similarly help trace the cellular origins of cfDNA released into blood, while plasma DNA methylation mapping and tissue-specific methylation markers further support cfDNA-based inference of tissue origin and cell death in contexts such as cancer, transplantation, and tissue injury [114,115,116]. DNA methylation connects molecular cell identity with measurable changes in tissue composition.

Overall, DNA methylation defines cell identity through regulatory stability, lineage-specific patterning, and cell-type-specific signatures. Its stability helps preserve somatic transcriptional programs and tissue homeostasis, and its plasticity allows methylation landscapes to shift during cell-state remodeling [117]. Abnormal deviations from lineage-associated methylation patterns can indicate altered cellular states and provide a basis for disease-related cell-state assessment [118,119].

### 3.2. Epigenetic Aging and Biological Clocks

Epigenetic clocks translate age-associated CpG methylation changes into quantitative estimates of biological age, providing a molecular framework for measuring aging beyond chronological time [120,121]. Unlike chronological age, biological age reflects variation in tissue functional decline, damage accumulation, and systemic homeostatic decline, making it more closely related to physiological aging and disease risk [120]. This approach is supported by large-scale methylome analyses showing that aging is accompanied by widespread, patterned, and increasingly variable CpG methylation changes [122]. DNA methylation clocks make aging measurable as a comparable molecular state, allowing age-related changes to be assessed across individuals, tissues, and health conditions [123].

Epigenetic aging varies across biological scales and forms of measurement. At the organ-system level, a single peripheral blood DNA methylation assay could estimate the aging status of 11 physiological systems, revealing that different systems age at different rates [124]. At the clinical outcome level, DNAm PhenoAge improved the prediction of aging-related morbidity, mortality, and physical functioning, while DNAm GrimAge further integrated methylation surrogates of plasma proteins and smoking exposure to predict lifespan and healthspan [125,126]. Beyond static age estimation, DunedinPACE measures the pace of biological aging from blood DNA methylation, making it useful for tracking aging rate over time [127]. At a finer biological resolution, single-cell methylation clocks reveal substantial cell-to-cell variation within the same individual, while studies of long-lived populations link healthier aging to younger methylation states and restrained increases in methylation entropy [128,129]. Epigenetic age can be read across several dimensions, including system-level divergence, clinical risk, aging rate, cellular variation, and entropy-related changes in methylome organization.

Epigenetic clocks quantify differences in biological aging across individuals, tissues, organ systems, and cell populations, but their interpretation depends on model construction, tissue-specific methylation baselines, and cellular composition. Clock-derived age should therefore be treated as a context-dependent estimate [130]. Their value lies in linking patterned age-associated methylation changes with both general aging processes and individual differences in physiological decline.

### 3.3. Circulating DNA Methylation as a Disease Biomarker

Cell-free DNA (cfDNA) methylation provides a disease biomarker that is both noninvasive and informative about tissue origin [131]. Sequence-based assays mainly capture genetic alterations, whereas cfDNA methylation retains epigenetic information from the cells that released the DNA. This makes it useful for early detection, prognosis, and tracing the tissue source of disease-associated signals [132]. Mechanistically, the strong disease-discriminating power of cfDNA methylation arises because methylation states are closely linked to chromatin packaging, gene expression, and fragmentation patterns [133].

The clinical value of cfDNA methylation can be understood through three main applications: broad cancer detection, tissue-of-origin prediction, and disease monitoring. For multi-cancer detection, the Circulating Cell-free Genome Atlas (CCGA) study showed that a targeted methylation-based test could detect cancer signals across more than 50 cancer types and predict cancer signal origin with high accuracy [134]. Comparative analyses of cfDNA features further support methylation as one of the most informative signals for multi-cancer early detection, particularly when benchmarked against mutation, copy-number, and fragmentation-based features [135]. Technical advances such as cfMethyl-Seq have also improved the feasibility of cost-effective cfDNA methylome profiling for cancer detection and localization [136]. For tissue localization, Conway et al. developed the cancer of unknown primary identification (CUPiD) classifier across 29 tumor types, achieving an overall sensitivity of 84.6% and a tissue-of-origin prediction accuracy of 96.8% in the validation cohort, with high concordance in patients with cancers of unknown primary [137]. Low-depth cfDNA methylation approaches that combine tumor-specific methylation atlases with genome-wide methylation density features further support the scalability of tissue-of-origin prediction [138]. Multimodal models extend this application by combining methylation and fragmentation features, as illustrated by THEMIS for multicancer detection and localization [139].

Multimodal diagnostic strategies can extend beyond the combination of cfDNA methylation and fragmentation features. Marchi et al. assembled the Acute Leukemia Methylome Atlas from 3314 leukemia samples across 11 harmonized cohorts and used machine learning models for subtype classification and prognostic assessment. Their workflow from specimen collection to clinical result applied native nanopore sequencing to profile DNA methylation together with single-nucleotide variants, structural variants, copy-number alterations, and short tandem repeats in the same sequencing run. Although this study analyzed leukemia specimens rather than cfDNA, it illustrates a broader diagnostic framework in which cohort harmonization and joint genomic and epigenomic analysis can translate complex methylation patterns into clinically relevant classification and risk stratification [140].

In colorectal cancer, cfDNA methylation has been used for both screening and treatment monitoring. MethyDT achieved an overall sensitivity of 91.2% and a specificity of 92.4%. After curative surgery, all positive cases became negative, suggesting that the methylation signal can reflect tumor removal. Similar evidence has come from treatment monitoring studies, where cfDNA methylation profiles were able to track response even when mutation data from the primary tumor were unavailable [141,142]. Post-treatment methylation signals may also inform minimal residual disease and recurrence risk. For instance, detection of methylated *BCAT1/IKZF1* after curative-intent treatment has been associated with poorer recurrence-free survival in colorectal cancer [143]. This monitoring strategy has also been explored in other cancers. In ALK-positive non-small cell lung cancer, longitudinal plasma cfDNA methylation profiling has been linked to prognosis and therapy monitoring [144]. Serial methylation-based circulating tumor DNA (ctDNA) assays are used to evaluate responses to anti-programmed cell death protein 1 (PD-1)-based immunotherapy in non-small cell lung cancer [145]. Together, these studies show that cfDNA methylation analysis can be used beyond diagnosis and tissue localization, with potential value in treatment-response assessment.

cfDNA methylation can reflect disease-associated epigenetic remodeling beyond conventional DMR patterns. In plasma cfDNA, differentially hemi-methylated regions (DHMRs) are largely distinct from differentially methylated regions (DMRs). Combining DHMR and DMR features improves the discrimination of healthy controls, liver cancer, and brain cancer [146]. This finding indicates that cfDNA-based biomarkers can include multiple forms of methylation alteration associated with disease state. Representative clinical applications of cell-free DNA methylation in cancer detection, tissue-of-origin prediction, and disease monitoring are summarized in Table 3.

**Table 3:** Representative clinical applications of cfDNA methylation analysis as disease biomarkers.

Application	Research Method	Analytical Feature	Key Performance	Clinical Scenario	Clinical Value	References
Broad cancer detection	Targeted methylation-based test (CCGA study)	Targeted cfDNA methylation signatures	Detects signals from >50 cancer types and predicts cancer signal origin with high accuracy	Population MCED screening	Early detection and signal localization	[134]
	Comparative cfDNA feature analysis in CCGA	Methylation vs. mutation, CNA, and fragmentation features	Methylation-related features rank among the most informative signals for MCED	Liquid biopsy strategy comparison	Supports methylation-centered MCED design	[135]
	cfMethyl-Seq	Enrichment-based cfDNA methylome profiling	Enrichment improves feasibility; multimodal integration strengthens detection and localization	Translational cancer screening assays	Cost-effective and integrated testing	[136]
Tissue-of-origin prediction	CUPiD classifier	Cancer-specific cfDNA methylation patterns	Reports 84.6% sensitivity and 96.8% prediction accuracy	CUP and lesion localization	Infers likely primary site	[137]
	Low-depth cfDNA methylation model	Tumor-specific methylation atlas & genome-wide methylation density	Supports tissue-of-origin prediction even with low-depth sequencing data	Clinically scalable tissue localization	Reduces technical burden for localization tasks	[138,139]
	THEMIS multimodal model	Methylation & fragmentation features	Enables simultaneous cancer detection and origin tracing	Cancer classification and lesion source identification	Joint detection and localization	[139]

cfDNA methylation makes it possible to detect disease-related epigenetic signals in circulating samples while also providing information about tissue origin and disease-associated methylation changes. However, clinical interpretation is still limited by low signal abundance, background noise, and sample heterogeneity, which reduce sensitivity and reproducibility [131]. The problem is especially clear in early-stage disease, where the tumor fraction is usually low. Pre-analytical variation and cohort heterogeneity add further uncertainty to diagnostic performance [147,148]. The plasma signal is also shaped by uneven ctDNA release. Apoptosis, necrosis, active secretion, tumor size, anatomical location, and vascularity can influence the amount of tumor-derived DNA that enters the circulation [149,150]. A plasma methylation profile may therefore preferentially represent regions with greater DNA release while underrepresenting spatially restricted clones. This can obscure disease-associated variation linked to progression, treatment resistance, or residual disease [151]. The resulting bias can complicate tissue origin assignment, molecular subtype classification, response assessment, and minimal residual disease monitoring. The spatial methylation approaches discussed in Section 3.4 can help identify the regional and clonal sources of circulating signals, placing cfDNA methylation results within their tissue context. Even with these constraints, cfDNA methylation remains useful as a systemic readout of disease presence, tissue origin, and epigenetic dysregulation.

### 3.4. Spatial and Regional Methylation Heterogeneity

cfDNA integrates methylation signals released from multiple tissue regions, but it cannot fully resolve their local origins or how epigenetic changes are organized within tissue environments [152]. Methylation patterns linked to disease are rarely uniform across a tissue. They shift between regions as cell composition, regulatory activity, and pathological remodeling vary across local environments [153]. Some differences arise from stable cell identity programs. Others develop as tissue maintenance, developmental history, or disease progression changes the local regulatory state [154,155]. Spatial methylation variation is therefore closely tied to how tissue niches acquire and maintain their distinct cellular features.

Mechanistically, local heterogeneity explains why methylation markers can support source tracing and tissue localization. Spatial methylation heterogeneity often arises from uneven local pressures within a tissue. In addition to cellular composition, oxygen supply, inflammatory signals, metabolic conditions, and cell communication also vary by region. These factors can reshape chromatin accessibility and transcription factor activity, creating regional methylation differences [156]. In tumors and other diseased tissues, this variation is further amplified by genetic alterations and by microenvironmental signals that promote epigenetic plasticity [157]. DNA methylation is also involved in immune remodeling and local immune evasion, connecting regional epigenetic variation with disease progression [158].

Spatial and region-resolved methods make methylation biomarkers easier to interpret within tissue context. Direct spatial methylation profiling methods, including spatial-DMT, can co-profile DNA methylation and transcription at near-single-cell resolution in the same tissue section [159]. This allows regional methylation differences to be compared with local gene expression profiles. Other spatial epigenome–transcriptome approaches integrate chromatin accessibility, histone marks, and gene expression, placing these molecular signals within tissue architecture [160]. In tumors, integrative spatial methods such as Tumoroscope link pathological images with genomic variation and spatial transcriptomic data. This makes it possible to map where different clones are located and to infer expression patterns associated with each clone [161]. Methylation-based regional analysis offers a related view. In microdissected colorectal tumors, local methylation profiles can be used to estimate epigenetic heterogeneity within the same tumor [162]. These approaches move methylation biomarkers beyond simple disease detection, allowing them to support tissue-of-origin inference and interpretation within spatial context.

**Figure 3:**
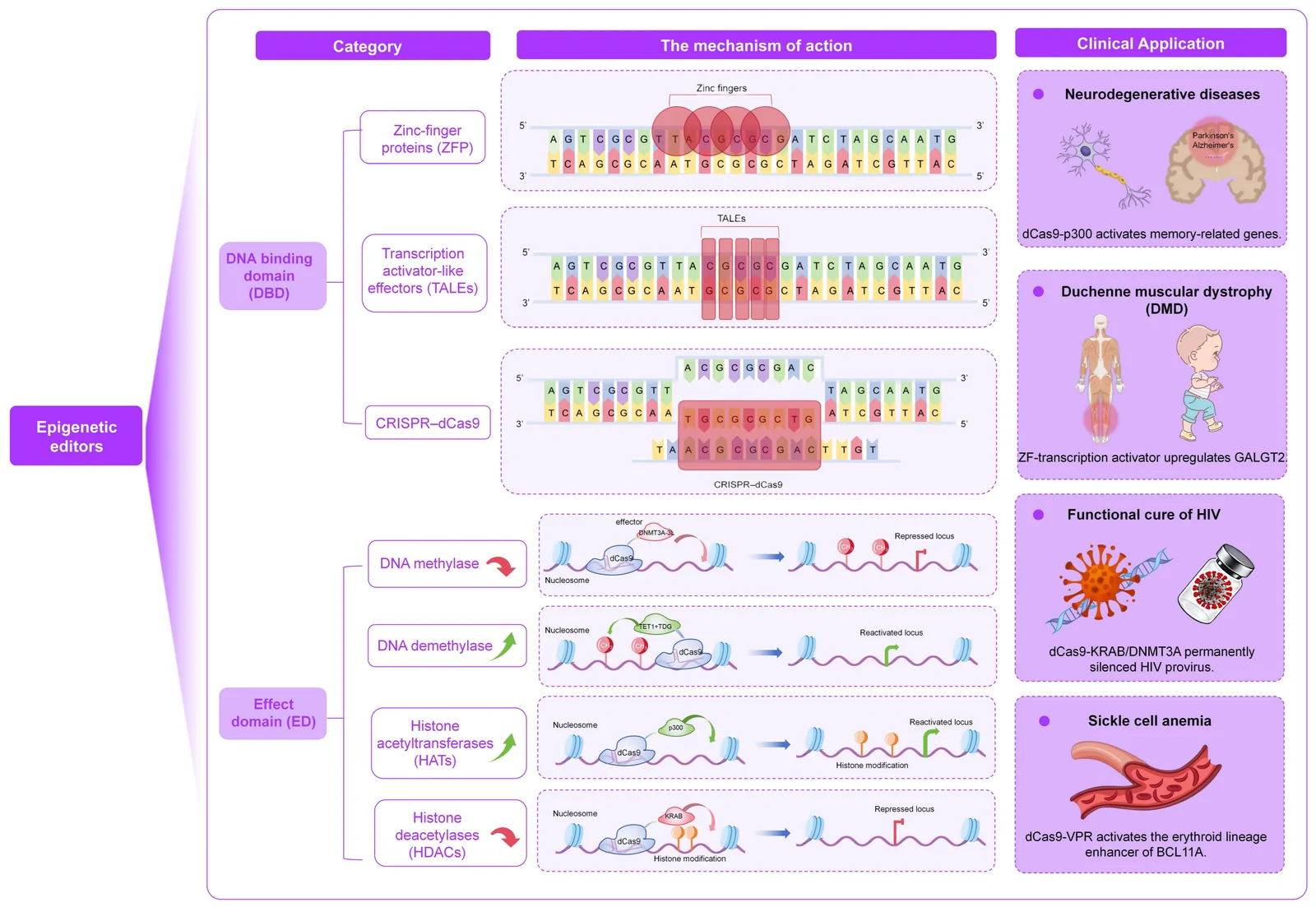
DNA methylation as a cellular and disease state indicator. DNA methylation functions as a multi-scale indicator of cellular identity, biological aging, and disease state. Cell-type-specific methylation signatures serve as stable epigenetic fingerprints that distinguish different cellular lineages. Age-associated methylation changes further enable the estimation of biological age and organ-system aging divergence through epigenetic clocks. In circulation, cfDNA methylation profiles provide noninvasive biomarkers for multi-cancer detection, tissue-of-origin prediction, and disease monitoring. At the tissue level, spatial methylation heterogeneity reflects local differences in cellular composition, hypoxia, inflammation, and tumor-associated remodeling, linking epigenetic variation to disease progression and tissue architecture. The figure was designed and assembled by the authors. Some graphical elements and icons were obtained from MedPeer (https://medpeer.cn/) and Iconfont (https://www.iconfont.cn/).

Spatial methylation heterogeneity connects methylation as a molecular mark with methylation as a disease biomarker. By linking regional methylation differences with cellular composition, regulatory programs, immune remodeling, and clonal organization, spatial methylation analysis provides information that cannot be fully resolved from bulk or circulating DNA profiles [163]. At present, spatial methylation approaches are still limited by resolution, cost, sample preservation, and computational standardization [164,165]. These constraints do not weaken the biomarker rationale. Instead, they show that methylation signals need to be interpreted with attention to where they come from and how they are measured. This is especially important because methylation profiles retain information about cell identity, disease-related remodeling, and tissue location. Under such circumstances, DNA methylation extends from a general marker of cell identity and disease state to a context-dependent readout of local tissue organization. The DNA-binding and effector components of epigenetic editors, together with their mechanisms of action and representative clinical applications, are summarized in Figure 3.

## 4. Programmable Epigenomes and Causal Inference

A central question in epigenetics is whether abnormal DNA methylation directly contributes to disease progression or accompanies changes in cellular and disease states. Epigenome-wide association studies (EWASs) have identified widespread associations between DNA methylation, complex diseases, aging, and cell fate determination [166,167]. However, such observations do not establish a direct causal relationship.

Genome editing platforms such as CRISPR/Cas9 introduce targeted sequence changes through programmable DNA recognition and have been explored for the treatment of cancer and other complex diseases [168,169]. Although these gene editing therapies are promising, challenges such as off-target effects, induced mutations, cytotoxicity, and long-term safety remain unresolved [170]. Most importantly, there are also risks such as chimeric phenomena and permanent sequence changes in embryos or in vivo applications, causing ethical issues [171]. Epigenome editing technology, without altering the DNA sequence, provides new possibilities for the treatment of various genetic and acquired diseases.

### 4.1. Epigenome Editing for Functional Validation

**Figure 4:**
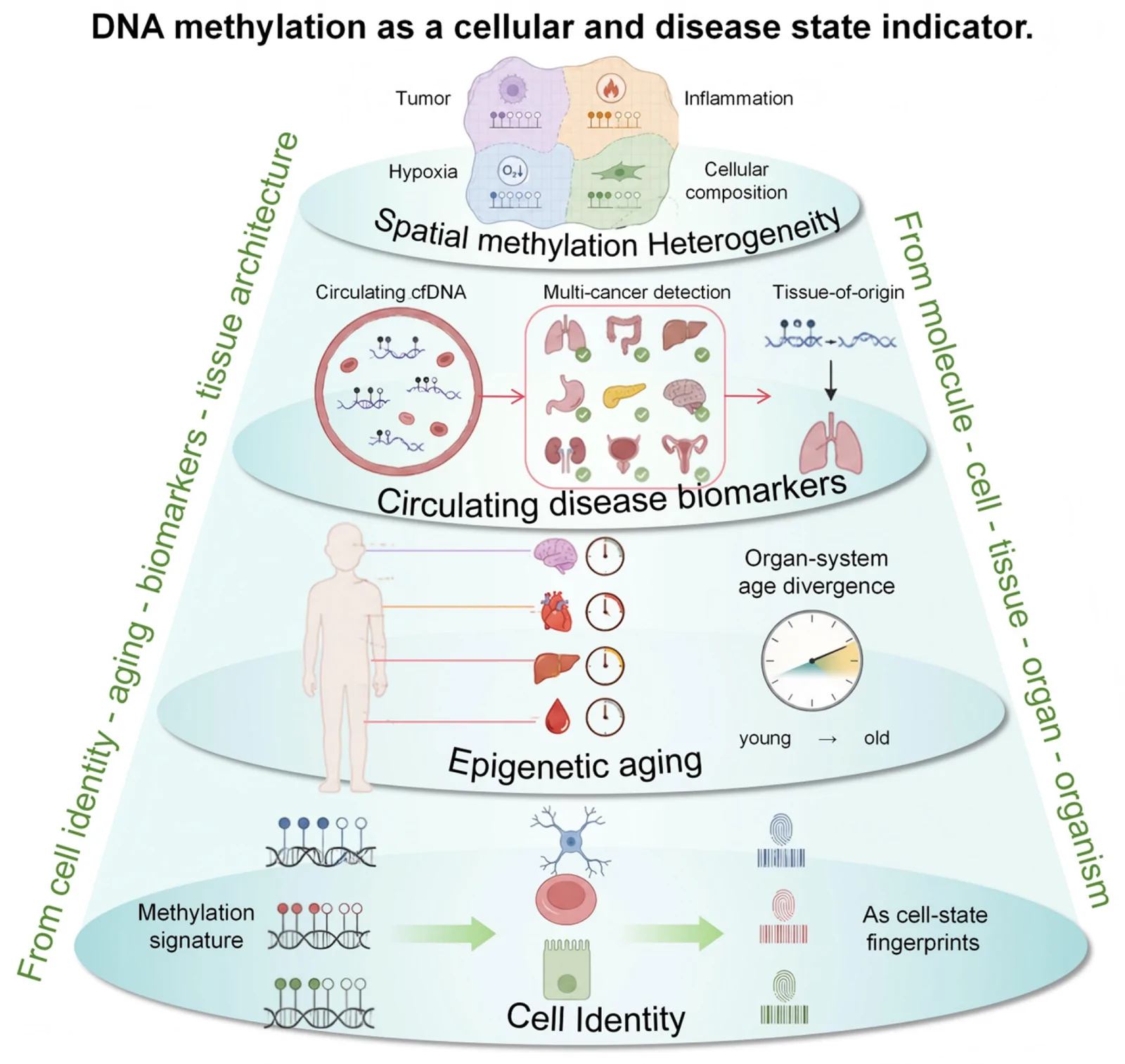
DNA methylation as a cellular and disease state indicator. The epigenome editing system comprises two functional modules: a DNA-binding domain and an effect domain. The DNA-binding domain consists of zinc-finger proteins, transcriptional activator-like effectors, and CRISPR-dCas9. Each of them targets the editing tool to specific genomic sites through distinct recognition mechanisms. The effect domain is responsible for catalyzing chromatin modifications. DNA methyltransferases and HDACs are employed for transcriptional repression, while DNA demethylases and HATs are used for transcriptional activation. These programmable systems have great potential in the clinical applications of neurodegenerative diseases, Duchenne muscular dystrophy, HIV silencing, and sickle cell disease. The figure was designed and assembled by the authors. Some graphical elements and icons were obtained from MedPeer (https://medpeer.cn/) and Iconfont (https://www.iconfont.cn/).

Epigenome editing uses programmable molecular tools to alter chromatin or DNA modifications at defined genomic loci [172]. These systems generally contain two functional components, a sequence-specific targeting module and an effector module that installs or removes an epigenetic modification [173]. To establish the causal relationship between DNA methylation and transcriptional regulation as well as cellular phenotype, we must understand how epigenetic marks are precisely written and erased. The roles of DNA methylation in cell identity, epigenetic aging, circulating disease biomarkers, and spatial methylation heterogeneity across cellular, tissue, and organismal scales are summarized in Figure 4.

The complete epigenome editing process is as follows. The catalytically inactive catalytically inactive CRISPR-associated protein 9 (dCas9) protein is first delivered to the promoter or enhancer region of the target gene by a pre-designed single-guide RNA (sgRNA) [169,174]. Subsequently, specific catalytic enzyme domains are recruited to target sites through direct fusion or non-covalent recruitment. Among these enzyme domains, the enzymes responsible for methylation writing are typically DNMT3A or work in collaboration with DNMT3L [175,176]. DNA demethylation is mediated by the dual oxidase catalytic domain of TET1, which converts 5mC to 5hmC and subsequently to unmodified cytosine through iterative oxidation [177,178]. Finally, these recruited enzymes interact with the local chromatin environment, thereby influencing epigenetic and transcriptional outputs. The principal DNA-binding and effector domains used in epigenome editing, together with their advantages, limitations, and application scenarios, are summarized in Table 4.

**Table 4:** Epigenome editing tools: the DNA-binding domain and the effect domain.

Epigenetic Editors	Edit Tools		Advantages	Disadvantages	Application Scenarios	References
DNA-binding domain (DBD)	Zinc-finger proteins (ZFP)		Compact in size and excellent bonding performance	Poor targeting ability	Clinical-grade applications	[179,180]
	Transcription activator-like effectors (TALEs)		Easier to assemble and highly targeted	Highly susceptible to 5-mc and unsuitable for use in mammals	Targeting of repetitive sequence regions	[181,182]
	CRISPR–dCas9		Simple design with multiple targeting options	Large volume and difficult delivery	High-throughput screening	[183,184,185]
Effect domain (ED)	Transcriptional activation (CRISPRon)	Ten–eleven translocation (TET) enzymes	Intermediate product 5hmC possesses independent functionality	Lower efficiency than DNA methylation	N-demethylation scenario	[186,187]
		Histone acetyltransferase (HAT)-p300	Powerful rapid activation	Unstable and reversible modifications	Enhancer Activation and Chromatin Openness Scenarios	[188]
		Thymine DNA glycosylase (TDG)	Synergistic enhancement with TET enzyme	Low efficiency, requiring cofactors	Reversing pathological silence	[33,189]
	Transcriptional repression (CRISPRoff)	DNA methyltransferase (DNMT3A-3L)	Maintained across multiple generations in stem cells	The binding site requires a CpG-rich region	Heritable Long-term Epigenetic Silencing Scenarios	[190]
		Histone deacetylases (HDACs) -Kruppel-associated boxes (KRABs)	The most potent transcriptional repression domain with sustained inhibitory effects	Reversal difficulty	Fast and Stable Suppression Scenario	[191,192]

Based on the aforementioned toolset, the combination of DNA-binding domains and effector domains can be regarded as controllable variables. Epigenome editing not only alters methylation states but also provides a controllable perturbation framework for establishing causal relationships between molecular events and phenotypes.

In causal inference of epigenome editing, functional validation on disease models is particularly critical. For example, in oncology, demethylation of the promoter regions of many tumor suppressor genes has been extensively reported. Targeted demethylation enables researchers to verify whether the removal of high methylation at specific sites is sufficient to reactivate silenced tumor suppressor genes, thereby preventing tumorigenesis [193]. Conversely, leveraging the promoter hypermethylation mediated by chimeric dCas9-Dnmt3a–Dnmt3L in normal cells can verify whether these epigenetic variants serve as direct pathogens that silence tumor suppressors and induce tumorigenesis [176]. Typically, experimental causal inference is designed according to a core framework comprising randomization, control, time series, dose, orthogonal validation, single-cell/family lineage analysis, and in vivo modeling [194]. A causal evidence chain is established through observable outcomes such as alterations in methylation levels or transcriptional phenotypic changes. Causal strength is classified into two categories based on the completeness of the causal chain and the depth of verification. Strong causality refers to the validation of the entire chain ‘editing–methylation alteration–target gene expression–functional phenotype–in vivo models’, with major confounding factors excluded through control experiments [195].

Epigenome editing has been applied in various disease models, including neurodevelopment, immunity, cardiovascular and central nervous system disease models, revealing how targeted methylation or demethylation establishes causal relationships at the molecular level. Table 5, below, summarizes the specific editing platforms, methylation directions, causal strength, and epigenetic stability of epigenomic therapies across various disease models.

### 4.2. Locus-Specific Methylation Perturbation

Numerous EWAS studies have revealed associations between DNA methylation and disease states. However, verifying whether these associations are causal requires intervention at the perturbation level [202]. Traditional perturbation methods, such as overexpression systems or DNMT inhibitors, have significant limitations. In vitro-generated overexpression or knockdown models cannot alter the methylation status of specific loci within the endogenous genomic environment [203]. Although the use of DNMT inhibitors can globally alter methylation levels and has been approved for clinical treatment, their nonspecificity severely limits causal inference [204]. One of the main advantages of epigenome editing technologies is that they can accurately add or remove methylation marks at specific DNA sites, so that the methylation state can be studied as an independent experimental variable while leaving the genome sequence unchanged [205].

However, from developing relevant tools to achieving reliable in vivo applications, this technology still faces three core challenges: how to minimize off-target effects while maintaining targeting efficiency, how to deliver molecular tools specifically to the desired cell populations within mixed tissues, and how to precisely control temporal and spatial perturbations in living cells.

#### 4.2.1. Challenges and Solutions for Targeting Precision

High-precision targeting is the prerequisite for locus-specific methylation perturbation. However, this objective faces multifaceted challenges in practice. For example, the dCas9 methyltransferase exhibits widespread off-target effects across the entire genome. The off-target sites of the fusion protein dCas9-DNMT3A/B are enriched in highly sensitive regions such as promoters and repetitive sequences, posing a potential threat to genomic stability [206]. Furthermore, a comparison of the performance of different epigenetic editors reveals that even CRISPRoff, which exhibits high targeting efficiency, induces persistent transcriptomic alterations [207]. Moreover, even when paired with non-targeted guide RNA (gRNA), CRISPRoff induces extensive methylation deposition and persistent transcriptomic alterations independent of methylation [208]. This indicates that off-target effects are not solely determined by gRNA specificity. The effector itself may possess a certain degree of nonspecific catalytic activity. In summary, the specificity issue in epigenome editing is not simply a binary distinction between off-target and on-target effects, but rather a multidimensional trade-off involving trade-offs among bystander modifications, chromatin environment dependence, and long-term transcriptional memory.

The low-level methylation alterations caused by off-target effects are easily overlooked but may lead to severe phenotypic consequences upon long-term accumulation. To enhance targeting accuracy, multiple engineering strategies have been proposed to date. For example, researchers developed a high-fidelity Cas9 variant with significantly reduced off-target effects through structure-guided engineering targeting the nonspecific interaction between the Cas9 protein and the DNA phosphate backbone. Based on the same DNA-binding interface, this finding also provides a reference for enhancing the targeting specificity of the dCas9 fusion protein [209,210]. Furthermore, the chemically induced system separates the DNA targeting module from the methylation catalytic module, with both components assembling into a functional complex only in the presence of small-molecule inducers. This design ensures that once DNA methylation is completed, the sustained activity of the effector ceases upon removal of the inducer, thereby reducing the risk of off-target accumulation [211].

A different strategy is to induce demethylation without directly tethering a broadly active TET catalytic domain to the target locus. Conventional dCas9-TET1 systems can reactivate methylation-silenced genes through locus-specific demethylation, but their editing range and specificity remain dependent on the recruited catalytic effector [212]. CRISPR-mediated DNA methylation interference (CRISPR-DiR) instead interferes with DNMT1-dependent methylation maintenance within a restricted genomic region and engages endogenous demethylation processes [213]. This design produces localized methylation loss while limiting changes in surrounding regions. It also enables methylation-sensitive regulatory elements to be linked with local and distal chromatin reorganization, which is useful for examining long-range regulatory interactions [213,214].

#### 4.2.2. In Vivo Delivery: Packaging Constraints and Cell-Type Selectivity

The transition from in vitro editing to in vivo application hinges on resolving two delivery challenges: payload size limitations and cell-type specificity. Most epigenome editing tools have large cargo sizes, exceeding the approximately 5 kb packaging limit of adeno-associated virus (AAV), which impedes efficient in vivo delivery [215]. The second challenge concerns how to achieve selective editing of specific cell subpopulations within mixed cell populations is also a critical factor determining their applicability in vivo. Substantial progress has been made on these two issues in recent years.

To enable the delivery of large genome editing tools, the split-Cas9 system overcomes this size limitation. The Cas9 effector fusion protein is divided into N-terminal and C-terminal fragments, each fused to the corresponding domain of the cleavage protein. The complete Cas9 protein is only reconstructed when co-expressed [216]. Another strategy circumvents viral vectors by combining an optimized compact mRNA structure with lipid nanoparticles (LNPs) for delivery. This enables efficient catalytic activity in vivo, where transient expression alone can reshape methylation states [217].

The second challenge pertains to cell-type specificity within heterogeneous tissues. Since disease-associated methylation states typically occur only in specific cell subpopulations, indiscriminate editing across all tissues may compromise therapeutic efficacy and safety. Therefore, emerging strategies employ cell-type-specific promoters and receptor-targeting nanoparticles to enhance spatial precision. The achievement of cell-type-specific editing of H3K9ac in intermediate progenitor cells (IPCs) during mouse cortical development demonstrates the feasibility of epigenome editing advancing from the ‘cell population level’ to the ‘specific cell subpopulation level’ [218].

#### 4.2.3. Temporal and Context-Dependent Control of Methylation Perturbation

Although programmable epigenome editing enables site-specific regulation of DNA methylation to the greatest extent possible, the same edit may still produce different outcomes. Increasing evidence indicates that the outcomes of epigenome editing are highly dependent on the chromatin environment and cellular state. At the same locus, identical methylation perturbations may yield distinctly different transcriptional outcomes depending on variations in CpG density, transcription factor composition, nucleosome accessibility, replication timing, and developmental stage [219,220]. Differences in editing efficacy may also reflect target selection. Some methylated regions correlate with gene repression, yet only a subset appears to exert direct causal effects on transcription. Broad promoter CpG islands are often chosen as editing targets, although functional methylation-dependent regulation is not confined to canonical CpG-island boundaries [18]. The proposed ‘Methylation Mesa’ model identifies narrow and discrete regulatory elements that are highly responsive to demethylation and may exert stronger effects on transcription than the surrounding methylation landscape [213]. Targeted demethylation of these elements produced greater transcriptional activation than editing the corresponding promoter CpG islands and was associated with local and distal chromatin reorganization. Editing a broad methylated region while missing such a focal element may therefore explain weak or inconsistent transcriptional responses. Target selection should consider functional regulatory evidence, local sequence context, and chromatin organization rather than CpG density alone [213,221].

Under different spatial contexts, promoters, enhancers, heterochromatin regions, and transcriptionally active sites with CpG enrichment in the local chromatin environment exhibit significantly distinct sensitivities to *de novo* methylation or demethylation. For example, the binding of transcription factors can interfere with DNMT3A recruitment through steric hindrance effects, while nucleosome compression may restrict the accessibility of target DNA sequences to dCas9-based editing tools [178]. Similarly, the interaction between DNA methylation and histone modifications is another critical factor. Regions with active transcription, a high degree of histone acetylation, and open chromatin are usually not prone to stable methylation deposition. The reason is that the endogenous transcriptional machinery continues to resist epigenetic inhibition [222,223]. Epigenome editing is also regulated by DNA sequence motif. For example, the study by Hackett revealed that even under the same chromatin modification strategy, the incorporation of H3K4me3 in certain promoters can guide transcription through hierarchical chromatin remodeling. In other promoters, however, the same modification produces attenuating or switch-like effects [224]. These findings demonstrate that the output of programmable methylation-editing phenotypes is spatially regulated by the surrounding chromatin ecosystem.

Temporal dynamics also influence the outcomes of epigenetic modifications. In previous experimental systems, while increasing the recruitment time of DNMT or TET effectors could boost the efficiency of editing, it also led to an increased risk of accumulation of off-targets and transcriptional instability. Researchers have developed different inducible regulatory networks that can regulate the length and timing of effector protein recruitment in an exact manner [225,226]. For example, the Chem-CRISPR/dCas9FCPF system allows accurate manipulation of the temporal window and dose of the effector domain through incorporation of the FCPF tag into dCas9 [227]. In recent years, optogenetic epigenome editing systems have further improved temporal precision. Light-responsive modules such as cryptochrome 2 (CRY2)-cryptochrome-interacting basic helix-loop-helix protein 1 (CIB1), light-oxygen-voltage (LOV), and phytochrome B (PhyB)-phytochrome-interacting factor (PIF) enable dynamic assembly and dissociation of dCas9-effector complexes, thereby achieving programmable epigenetic regulation with high spatiotemporal resolution [228].

### 4.3. Epigenetic Stability and Reversibility

The stable inheritance of DNA methylation relies largely on a set of maintenance mechanisms inside the cell. In the process of DNA replication, the enzyme DNMT1 is responsible for performing maintenance methylation, thereby ensuring faithful transmission of methylation states to daughter cells [229]. Epigenome editing exactly utilizes this endogenous maintenance mechanism. In many cases, the dCas9 effector functions primarily as a trigger and recruitment platform. Even in the absence of the editing factor, the endogenous chromatin regulatory system of the cell can still maintain heritable transcriptional memory [18]. For example, the ‘instant delivery and permanent retention’ model was successfully demonstrated in vivo by Cappelluti. Following a single injection of LNP-packaged CRISPRoff mRNA, the *PCSK9* gene in the liver cells of mice was silenced for about 330 days [199]. This finding suggests that epigenetic therapies have the potential to produce long-term therapeutic effects with the help of transient interventions. However, the stability of different epigenetic effectors varies depending on the effector employed. Histone acetylation-based activation systems often induce fast yet short-term transcriptional activation because histone acetylation is dynamically countered by endogenous histone deacetylases [188]. By contrast, DNA methylation modification systems, particularly those employing DNMT3A/3L fusion proteins or CRISPRoff platforms, tend to establish a more persistent silencing state. Such durability is especially evident at CpG-rich promoters and within chromatin environments associated with stem cell identity [185]. This difference suggests that not all chromatin modifications have the same genetic potential. Some modifications only act as transient signal intermediates, while others can be incorporated into the long-term chromatin memory system.

However, epigenetic stability does not equate to absolute irreversibility. The methylation status of the programmable epigenome is regulated by a dynamic balance between endogenous maintenance mechanisms and active clearance pathways. Even after methylation editing has been completed, the newly established epigenetic state can be disrupted by chromatin remodeling factors, DNA replication stress, inflammatory signal transduction, and TET-catalyzed oxidation [105,230,231]. In particular, in rapidly proliferating tissues or embryonic development stages, local methylation memory may gradually fade because of continuous chromatin turnover and cellular state transitions [232]. Thus, the long-term maintenance of epigenome editing outcomes largely depends on the interplay between genomic context and the cellular environment. Compared to permanent genome editing, reversibility is a major benefit of epigenetic engineering. Unlike the irreversible sequence modifications induced by nuclease systems, programmable methylation editing theoretically enables the reprogramming, updating, or elimination of disease-associated transcriptional states according to therapeutic needs [233]. For example, the CRISPRon and CRISPRoff platforms establish a bidirectional regulatory mechanism that can be switched between transcriptional silencing and activation through targeted methylation or demethylation [18]. This reversibility provides additional safety assurance for disease treatment, particularly in the fields of neurological disorders, developmental diseases, and regenerative medicine.

The ultimate challenge in epigenetic engineering lies not only in establishing methylation states, but also in regulating the balance between their stability and reversibility. Whether epigenetic therapies are designed to exhibit transient, persistent, or conditionally reversible temporal patterns, these features can be treated as programmable design parameters. This approach ensures both sufficiently durable therapeutic effects and the ability to maintain controllable reprogramming capabilities.

## 5. Outlook: Toward Epigenome Engineering

This review defines DNA methylation as a programmable information layer that operates at molecular, cellular, and clinical levels. From the chemical diversity of cytosine modifications in chromatin-restricted environments and their enzymatic regulation, to the application of methylation profiles as cellular identity markers and circadian clocks, to CRISPR-based epigenome editing platforms enabling the establishment of causal relationships between specific methylation events and disease-associated phenotypes, a clear developmental trajectory is gradually emerging. DNA methylation is evolving from a passive observation indicator to an actively controllable regulatory parameter. Studies have demonstrated that transient delivery of epigenome editing tools can achieve durable gene silencing/activation effects without permanent alterations to the genomic sequence. This makes epigenome editing a long-term therapeutic approach distinct from traditional gene-editing methods.

However, before programmable epigenomic technologies can be widely applied in clinical practice, numerous significant challenges remain to be overcome. Off-target methylation deposition, transcriptional outcomes influenced by local chromatin architecture, delivery limitations in heterogeneous tissues, and variations in long-term epigenetic stability across different cellular environments all require ongoing and in-depth research. In the future, it will be essential to integrate technologies such as inducible optogenetic control techniques and delivery systems capable of targeting specific cell types, thereby enabling DNA methylation to fully manifest its therapeutic potential as a designable regulatory mechanism. Ultimately, researchers will not only be able to diagnose disease states based on methylation profiles but also actively reprogram these disease states at the epigenetic level.

## Figures and Tables

**Table 1:** Scoring criteria for the evaluation of DNA methylation profiling technologies.

Dimensions	Definition	Interpretation of the 0–3 Scale
DIF	DNA integrity and fidelity	3 = native/minimally damaging readout 2–2.5 = low-damage enzymatic or chemical conversion 1–1.5 = damage-prone conversion or substantial fragmentation 0–0.5 = severe damage or indirect/low-fidelity signal
RLC	Read length and context	3 = long-read single-molecule context with haplotype/structural/regional linkage 2–2.5 = targeted or partial long-range context 1–1.5 = short-read or fragmented context 0–0.5 = little long-range information
MR	Modification resolution	3 = base-resolution and modification-specific discrimination 2–2.5 = strong 5-mC calling but limited separation of related marks 1–1.5 = combined or indirect 5-mC/5-hmC information 0–0.5 = low-resolution assessment
PAS	Protocol and analysis simplicity	3 = low-input, standardized, relatively simple workflow and analysis 2–2.5 = manageable workflow with some extra steps 1–1.5 = complex preparation, targeted enrichment, high depth, or model-dependent analysis 0–0.5 = highly specialized or poorly standardized workflow

**Table 5:** Functional validation of epigenome editing across disease models: causal evidence from targeted methylation to disease-relevant phenotypes.

Body System	Disease Model	Pathogenesis	Epigenome Editing Platform	Target Gene	Methylation Direction	Causality	Apparent Stability	References
Nervous system	Fragile X syndrome (FXS)	Amplified 5′ CGG repeat → high promoter methylation → silencing of the FMR1 gene	dCas9-Tet1/ sgRNA	*FMR1*	Demethylation	Strong causality	Several weeks	[196]
	Rett syndrome	*MECP2* is silenced on the inactivated X chromosome in females.	dCas9-Tet1 and dCpf1-CTCF	*MECP2*	Demethylation	Strong causality	Several weeks	[197]
Immune system	Treg dysfunction	Loss of FOXP3 expression → loss of Treg function	dCas9-TET1CD	Treg enhancer-TSDR and gene *FOXP3*	Demethylation	Conditional causality	Several months	[198]
Cardiovascular system	Hypercholesterolemia	Highly expressed *PCSK9* → Cholesterol receptor degradation → Elevated cholesterol LDL-C levels	ZFP-EvoETR	*PCSK9*	Methylation writing	Strong causality	Long-term maintenance	[199]
Central-nervous system	Glioblastoma	Highly expressed *MGMT* gene → MGMT repair protein → Tumor cells’ defense against chemotherapy	CRISPRoff	*MGMT*	Methylation writing	Strong causality	Multi-generation division stability	[200]
	Prion diseases	The highly expressed *PRNP* gene encodes misfolded prion protein (PrP).	CHARM	*PRNP*	Methylation writing	Strong causality	Long-term maintenance	[201]

## Data Availability

No new data were created or analyzed in this study. Data sharing is not applicable to this article.

## Abbreviations

The following abbreviations are used in this manuscript:

2-HG	2-hydroxyglutarate
5-caC	5-carboxylcytosine
5-fC	5-formylcytosine
5-hmC	5-hydroxymethylcytosine
5-mC	5-methylcytosine
AAV	Adeno-associated virus
ADD	ATRX-DNMT3-DNMT3L domain
ALK	Anaplastic lymphoma kinase
AP-1	Activator protein 1
APOBEC	Apolipoprotein B mRNA editing enzyme, catalytic polypeptide-like
AUC	Area under the receiver operating characteristic curve
BAM	Binary alignment/map
BER	Base excision repair
CCGA	Circulating Cell-free Genome Atlas
CCS	Circular consensus sequencing
cfDNA	Cell-free DNA
CGI	CpG island
CIB1	Cryptochrome-interacting basic helix-loop-helix protein 1
CNA	Copy-number alteration
CRC	Colorectal cancer
CRISPR	Clustered regularly interspaced short palindromic repeats
CRISPR-DiR	CRISPR-mediated DNA methylation interference
CRY2	Cryptochrome 2
CTCF	CCCTC-binding factor
ctDNA	Circulating tumor DNA
CUP	Cancer of unknown primary
CUPiD	Cancer of unknown primary identification
dCas9	Catalytically inactive CRISPR-associated protein 9
DBD	DNA-binding domain
DHMR	Differentially hemi-methylated region
DIF	DNA integrity and fidelity
DMR	Differentially methylated region
DNMT	DNA methyltransferase
DNMT1	DNA methyltransferase 1
DNMT3A	DNA methyltransferase 3A
DNMT3B	DNA methyltransferase 3B
DNMT3L	DNA methyltransferase 3-like
ED	Effector domain
EM-seq	Enzymatic methyl sequencing
EWAS	Epigenome-wide association study
FCPF	FCPF chemically responsive tag
FXS	Fragile X syndrome
GC	Guanine–cytosine
HAT	Histone acetyltransferase
HDAC	Histone deacetylase
HIV	Human immunodeficiency virus
IDH1	Isocitrate dehydrogenase 1
IDH2	Isocitrate dehydrogenase 2
IPC	Intermediate progenitor cell
IPD	Interpulse duration
iPSC	Induced pluripotent stem cell
KRAB	Krüppel-associated box
LDL-C	Low-density lipoprotein cholesterol
LNP	Lipid nanoparticle
LOV	Light-oxygen-voltage
LSH	Lymphoid-specific helicase
MCED	Multi-cancer early detection
MR	Modification resolution
MRD	Minimal residual disease
mRNA	Messenger RNA
nCATS	Nanopore Cas9-targeted sequencing
NSCLC	Non-small cell lung cancer
ONT	Oxford Nanopore Technologies
OSK	OCT4, SOX2, and KLF4
OSKM	OCT4, SOX2, KLF4, and c-MYC
PAS	Protocol and analysis simplicity
PCR	Polymerase chain reaction
PD-1	Programmed cell death protein 1
PhyB	Phytochrome B
PIF	Phytochrome-interacting factor
PWWP	Pro-Trp-Trp-Pro domain
PW	Pulse width
RLC	Read length and context
SAM	S-adenosylmethionine
sgRNA	Single-guide RNA
SMRT	Single-molecule real-time
SNV	Single-nucleotide variant
STR	Short tandem repeat
SV	Structural variant
SWI/SNF	Switch/sucrose non-fermentable
T4-BGT	T4 bacteriophage beta-glucosyltransferase
TAD	Topologically associating domain
TALE	Transcription activator-like effector
TAPS	TET-assisted pyridine borane sequencing
TAPSβ	Beta-glucosyltransferase-assisted TAPS
TCA	Tricarboxylic acid
TDG	Thymine DNA glycosylase
TET	Ten–eleven translocation
TRD	Target recognition domain
Treg	Regulatory T cell
TSDR	Treg-specific demethylated region
UBS-seq	Ultrafast bisulfite sequencing
UHRF1	Ubiquitin-like with PHD and RING finger domains 1
wglrTAPS	Whole-genome long-read TAPS
ZFP	Zinc-finger protein
βGT	Beta-glucosyltransferase
